# Managing therapeutic resistance in breast cancer: from the lncRNAs perspective

**DOI:** 10.7150/thno.49922

**Published:** 2020-08-18

**Authors:** Liyuan Peng, Jingwen Jiang, Bo Tang, Edouard C. Nice, Yuan-Yuan Zhang, Na Xie

**Affiliations:** 1 State Key Laboratory of Biotherapy and Cancer Center, West China Hospital, and West China School of Basic Medical Sciences & Forensic Medicine, Sichuan University, and Collaborative Innovation Center for Biotherapy, Chengdu, 610041, P.R. China.; 2 Department of Urology, Institute of Urology, West China Hospital, Sichuan University, Chengdu, 610041, P.R. China.; 3 Department of Biochemistry and Molecular Biology, Monash University, Clayton, Victoria, Australia.; 4 West China School of Basic Medical Sciences & Forensic Medicine, Sichuan University, Chengdu, 610041, P.R. China.

**Keywords:** Long noncoding RNA, Breast cancer, Drug resistance, Biomarkers, Therapeutic targets

## Abstract

Breast cancer (BC) is the most common female malignancy and the second leading cause of cancer-related death worldwide. In spite of significant advances in clinical management, the mortality of BC continues to increase due to the frequent occurrence of treatment resistance. Intensive studies have been conducted to elucidate the molecular mechanisms underlying BC therapeutic resistance, including increased drug efflux, altered drug targets, activated bypass signaling pathways, maintenance of cancer stemness, and deregulated immune response. Emerging evidence suggests that long noncoding RNAs (lncRNAs) are intimately involved in BC therapy resistance through multiple modes of action. Therefore, an in-depth understanding of the implication of lncRNAs in resistance to clinical therapies may improve the clinical outcome of BC patients. Here, we highlight the role and underlying mechanisms of lncRNAs in regulating BC treatment resistance with an emphasis on lncRNAs-mediated resistance in different clinical scenarios, and discuss the potential of lncRNAs as novel biomarkers or therapeutic targets to improve BC therapy response.

## Introduction

Breast cancer (BC) is one of the three most frequent cancers worldwide and also the most common cancer in females, with approximately one in ten women at risk of suffering it during their lifetime 1. Moreover, BC is the leading cause of cancer-related death in less developed countries and ranks second in more developed countries 1. Currently, the mainstay treatments of BC involve endocrine therapy, anti-human epidermal receptor 2 (HER2) target therapy and cytotoxic chemotherapy depending on individual BC subtypes. Based on emerging preclinical and clinical studies, combinational treatment of targeted drugs (e.g. mTOR inhibitor everolimus 2, cyclin-dependent kinase 4/6 (CDK 4/6) inhibitor palbociclib 3) with endocrine therapy has prolonged progression-free survival (PFS) in certain BC patients. These therapeutic strategies along with early screening tools (e.g. mammography 4, tomosynthesis 5 and magnetic resonance imaging (MRI) 6) have partly decreased BC mortality; however, the clinical therapeutic outcome is far from satisfied. The major barrier for complete BC cure is the development of therapeutic tolerance. BC cells are equipped with numerous mechanisms to cope with different treatment strategies, such as increased drug efflux, altered drug targets and activated bypass signaling pathways, maintenance of cancer stemness, as well as deregulated immune response. Therefore, there is still an urgent need for extensive research on BC therapy resistance to develop novel biomarkers and therapeutic targets that could predict therapeutic response or improve clinical outcomes of BC patients.

Basic research on BC therapeutic resistance has focused more on protein-coding genes as their products are thought to play a central role in regulating biological activities. However, only about 2% of the human transcriptome encodes proteins 7, while the remainder of the transcriptome has no obvious protein-coding potential (referred to as noncoding RNAs, ncRNAs). The past few years have witnessed exciting advances in the functional and mechanistic characterization of ncRNAs, especially long noncoding RNAs (lncRNAs) which contain the largest percentage of the noncoding transcriptome 8. Since the identification of the first lncRNA H19 in fetal liver tissue in 1900 9, thousands of lncRNAs have been found and investigated. Notably, an increasing number of lncRNAs have been found to be associated with pathological scenarios such as neurodegenerative disorders, cardiovascular diseases and cancers. For example, several lncRNAs identified in *HOX* gene foci have been shown to play essential roles in cancer initiation and development 10.

Although the specific functions and exact underlying mechanisms of most BC-related lncRNAs remain elusive, there have been increasing studies on dysregulated lncRNAs in BC therapeutic resistance (**Table 1**). For example, upregulated expression of lncRNA HOTAIR and nuclear paraspeckle assembly transcript 1 (NEAT1) are responsible for BC therapeutic resistance to endocrine therapy and chemotherapy, respectively 11-13. In particular, Cantile *et al.* have recently published a comprehensive review on the role of HOTAIR in BC therapy, which introduces numerous recent and influential studies 14. Furthermore, growing evidence has indicated the great potential of lncRNAs to serve as biomarkers to predict treatment response, such as HOXB-AS5 15, Lnc712 16 and the long intergenic non-coding RNA for kinase activation (LINK-A) 17. LncRNAs can also be applied as therapeutic targets to help tackle treatment resistance in BC. For example, therapeutic delivery of locked nucleic acids (LNAs) targeting LINK-A has been proven to confer BC sensitivity to immune checkpoint inhibitors in a preclinical study 17.

In this review, we will discuss biological functions and underlying mechanisms of dysregulated lncRNAs in BC therapy resistance with an emphasis on lncRNAs-mediated therapeutic resistance in BC clinical scenarios. Additionally, we also highlight the advantages and challenges lying ahead for the application of lncRNAs as biomarkers or targets for restraining treatment resistance in BC.

## LncRNAs-mediated BC therapeutic resistance

The mechanisms of resistance to different BC therapeutic strategies share many similarities, including increased drug efflux, altered drug targets, activated bypass signaling pathways, as well as maintenance of cancer stemness. Besides, deregulated immune response has also been identified as an essential contributor to BC immunotherapy resistance. Emerging evidence has demonstrated that lncRNAs participate in therapeutic resistance of BC through multiple modes of action. As the role of lncRNAs in increased drug efflux has been comprehensively reviewed elsewhere 18, here we focus on the molecular mechanisms of lncRNAs-mediated BC therapeutic resistance including the alteration of drug targets, downstream bypass pathways, cancer stemness, and immune response.

### LncRNAs in drug targets and bypass signaling alteration

Drug efficacy is largely determined by the protein level and mutation state of drug targets, as well as the activation of bypass signaling pathways. One example is the altered expression of estrogen receptor α (ERα), which is a target of endocrine therapy for BC. It has been reported that Notch and HGF signaling-mediated upregulation of lncRNA H19 could promote ERα expression at both mRNA and protein levels. Thus, H19 counteracts endocrine therapy-mediated downregulation of ERα protein and is responsible for therapeutic resistance in BC cells 19. Similarly, lncRNA MIR2052HG increases the transcription of the ERα encoding gene *ESR1* and reduces ERα degradation through LMTK3, thus resulting in resistance to endocrine therapy 20. LncRNA TINCR upregulated in BC cells sponges miR-125b to increase the expression level of HER2, resulting in the resistance of BC cells to anti-HER2 targeted therapy 21.

In addition to drug target alteration, lncRNAs are implicated in activation of bypass signaling pathways to mediate BC therapeutic tolerance. A typical example is the ligand-independent ERα activation that impedes the efficacy of endocrine agents. ERα can be phosphorylated by MAPK and AKT independent of the ligand binding, leading to endocrine resistance 22-24. According to a recent study, linc-RoR promotes ligand-independent cell growth through activating MAPK/ERK pathway and results in BC endocrine resistance 25. Taken together, these findings indicate that lncRNAs play essential roles in modulating therapeutic resistance by bypassing the original drug targets. Further investigations are required to unmask the lncRNAs-associated redundant pro-resistant signaling pathways involved in BC therapeutic strategies. Targeting fundamental lncRNAs in these redundant pro-resistant signaling pathways may exhibit promising effects in clinical situations.

### LncRNAs in cancer stemness maintenance

CSCs are considered as a self-renewing subpopulation of neoplastic cells among heterogeneous tumors and were first documented in 1997 in the hematopoietic system 26. Later in 2003, BC became the first solid tumor in which CSCs were discovered. The existence of breast cancer stem cells (BCSCs) poses a tremendous challenge for BC treatment due to their inherent therapeutic resistance to conventional therapies. Therefore, more in-depth research is required to explore the regulatory networks of BCSCs formation and maintenance.

A recent study reveals that lncRNA Peblr20 can enhance pluripotent reprogramming thus maintaining pluripotency of induced pluripotent stem cells (iPSCs) 27, implicating that lncRNAs may also orchestrate the preservation of cancer stemness. Previous studies have illustrated a direct nexus between epithelial-mesenchymal transition (EMT) and cancer stemness, especially in BC 28-29. Emerging studies have revealed that lncRNAs are vital novel players in the regulation of EMT-associated BCSC stemness 30-31. For instance, several lncRNAs have been demonstrated to maintain or enhance both EMT traits and CSC-like characteristics of BC cells, including LINC01638 32, lncRNA RP1 33, LINC-ZNF469-3 34 and LINC-ROR 35. A recent study provides a more detailed possible mechanism through which lncRNA-Hh upregulated by Twist directly targets the hedgehog signaling (Hh) enhancer GAS1, to activate Hh and increase the expression of Gli1, SOX2 and OCT4 for BCSC maintenance 36. The Twist-lncRNA-Hh-Hh-SOX2/OCT4 axis partly explains why epithelial BC cells with EMT phenotype also gain CSC-like properties. Recently, Tang *et al.* established the direct nexus between lncRNA-regulated EMT and resistance to BC therapy. According to their study, DCST1-AS1 enhances EMT and promotes TNBC chemoresistance to doxorubicin and paclitaxel by directly interacting with ANXA1 37. However, the underlying mechanism of DCST1-AS1-ANXA1 axis-mediated doxorubicin resistance remains to be further elucidated.

Furthermore, it has been well documented that some of the pluripotency factors (e.g. OCT3/4, SOX2, KLF4, LIN28) and CSC markers (e.g. ALDH1A3) 38-45 are capable of promoting stemness in BCSCs. Increasing evidence has implicated the pivotal role of lncRNAs in BCSCs maintenance through their interplay with these stemness-associated factors. Recently, Xu and colleagues have demonstrated that lncRNA CCAT2 enhances the expression of OCT4, Nanog and KLF4, as well as increases the ALDH^+^ CSC subpopulation in TNBC 46. In addition, a number of lncRNAs have been found to act as competing endogenous RNAs (ceRNAs), which compete against the limited microRNAs (miRNAs) pool, to regulate the expression of pluripotency factors and CSC markers. For example, highly expressed lncRNA H19 in BCSCs acts as a ceRNA to sponge miRNA Let-7, resulting in the increased expression of a Let-7 target LIN28 thus promoting the preservation of BCSCs 47. Intriguingly, LIN28 reversely inhibits Let-7 production and maturation, further de-repressing H19 expression in BCSCs 47. The positive feedback loop formed by H19, Let-7 and LIN28 maintains the stemness of BCSCs, indicating that the disruption of this axis may provide opportunities for reversal of treatment tolerance. Likewise, mesenchymal stem cells (MSCs)-induced lncRNA LINC01133 positively regulates KLF4 to promote phenotypic and growth characteristics of BCSCs 48. Moreover, lncRNA-Hh promotes the activation of the hedgehog signaling molecule Hh to upregulate SOX2 and OCT4 for BCSC maintenance 36. The lncRNA HOTTIP regulates the miR-148a-3p/WNT1 pathway to maintain the CSC-like properties of BCSCs and facilitate BC growth 49. Notably, NRAD1 was identified as the first lncRNA which can be activated by a CSC marker 50. Mechanistically, ALDH1A3 and its product retinoic acid positively regulate the expression of NRAD1, thus enhancing the interaction between NRAD1 and genes involved in differentiation and catabolism, eventually promoting cell survival and increasing the number of BCSCs 50. Taken together, lncRNAs are widely involved in BCSCs preservation and may lead to intrinsic therapeutic tolerance, however, the underlying mechanisms and clinical value remain to be thoroughly explored.

### LncRNAs in immune response deregulation

Cancer immunotherapy is an emerging treatment option taking advantage of the cytotoxic potential of the immune system. In spite of the encouraging progress in BC immunotherapy, cancer cells have been reported to develop numerous mechanisms to evade immune elimination, including reduced tumor antigenicity, increased activation-induced cell death (AICD) of T lymphocytes and re-activation of oncogenic signaling 51. For example, lncRNA LINK-A plays a central role in antigenicity loss and immune checkpoints evasion in BC through directly interacting with phosphatidylinositol-(3,4,5)-trisphosphate and inhibitory G-protein-coupled receptor (GPCR). Such interactions lead to reduced cyclic AMP (cAMP) concentrations and subsequent protein kinase A (PKA)-mediated TRIM71 phosphorylation. Consequently, phosphorylated-TRIM71 enhances proteasome-mediated degradation of peptide-loading complex (PLC) components, thus resulting in decreased antigen presentation to the surface of BC cells 17. Another independent study has revealed that lncRNA NKILA enhances T cell vulnerability to AICD by interacting with NF-κB. Thus, the apoptosis and subsequent reduced infiltration of cytotoxic T lymphocytes (CTLs) might contribute to immunotherapy resistance 52. Overall, lncRNAs are intimately related to deregulated immune response, thus conferring resistance to immunotherapy in cancer cells. Targeting lncRNAs may present a promising strategy to reverse therapeutic resistance and achieve better clinical outcome for BC patients.

## LncRNAs-mediated BC resistance in different clinical scenarios

**Figure 1:**
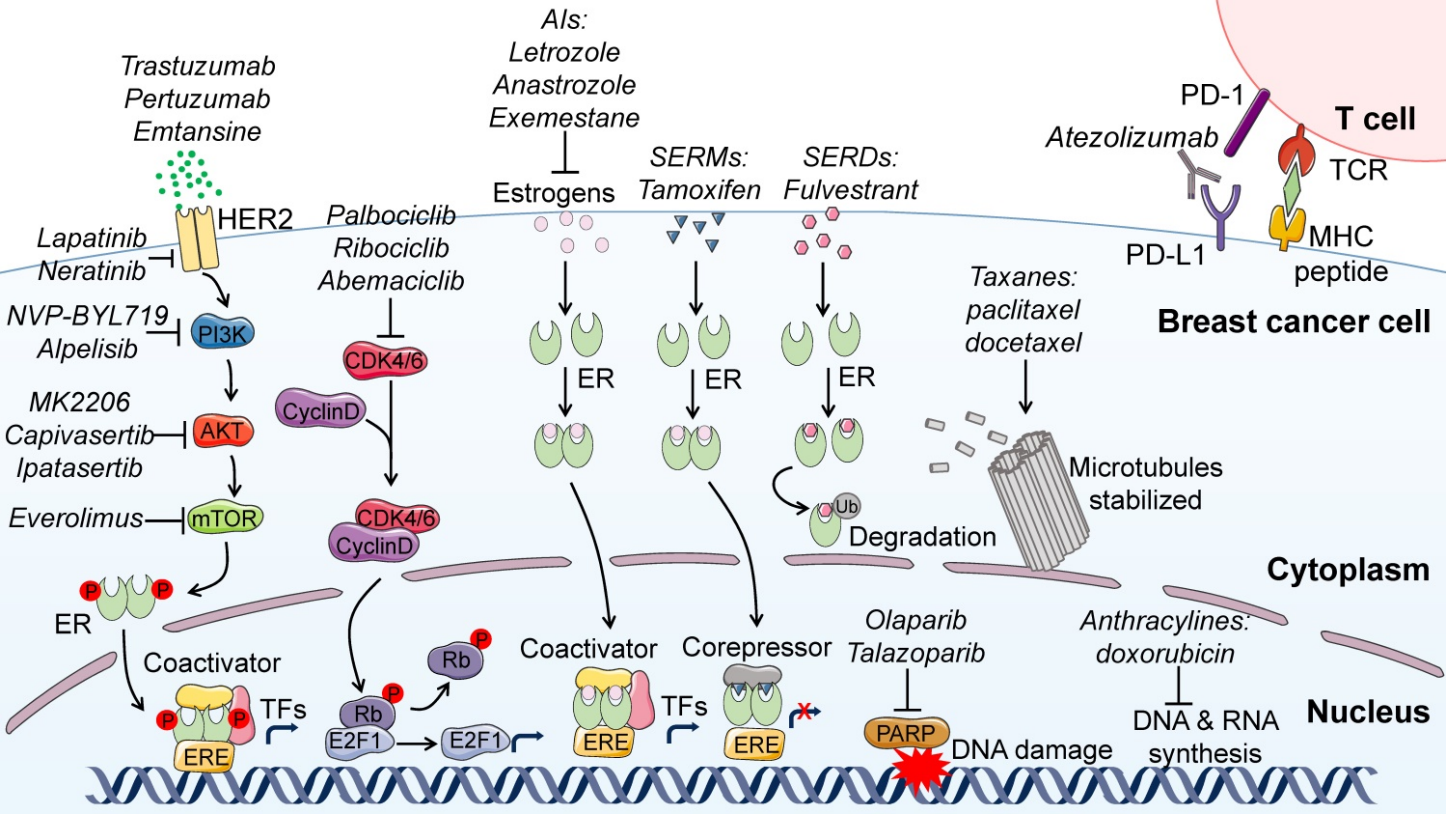
** Clinical therapeutic strategies for BC.** Currently, endocrine therapy, anti-HER2 targeted therapy and chemotherapy constitute the backbone of BC treatment in clinic. Drugs for endocrine therapies include selective estrogen receptor modulators (SERMs), selective estrogen receptor degraders (SERDs) and aromatase inhibitors. SERMs bind ER to antagonize the activity of estrogen, SERDs bind ER to promote its proteasome-mediated degradation, while aromatase inhibitors block the biosynthesis of estrogens from adrenal steroids. Drugs available for anti-HER2-targeted therapy include monoclonal antibodies (trastuzumab, pertuzumab and emtansine) and small molecules (lapatinib and neratinib). Chemotherapy standards for BC treatment are anthracylines and taxanes. In addition, novel therapeutic strategies including targeted therapies beyond HER2 and immunotherapy have been administered in clinical situations (drugs for BC treatment are shown in italic).

Based on the expression of specific biomarkers including ER, progesterone receptor (PR) and HER2, BC has been classified into at least four clinically relevant subtypes, including luminal A, luminal B, HER2-enriched, and basal like subtype. The two luminal subtypes are commonly characterized with positive ER or PR (or both) expression and negative HER2 expression, among which luminal A subtype is characterized with less proliferative potential. HER2 subtype BC is characterized by the overexpression of HER2, which can be subdivided into non-luminal (ER and PR negative) and luminal (ER or PR positive, or both) subtype. The basal-like subtype BC shows positive basal marker expression and usually negative expression of ER, PR and HER2, thus most of basal-like subtype BC is triple-negative breast cancer (TNBC) 1. Among the four main subtypes, the luminal A subtype shows favorable prognosis, while the basal-like subtype exhibits the most aggressive clinical behavior. Based on these different clinical subtypes of BC, endocrine therapy, anti-HER2 targeted therapy and chemotherapy constitute the backbone of BC treatment (**Figure 1**). These clinical mainstay strategies, along with immunotherapy and targeted therapies beyond HER2, have, to a large extent, improved the PFS of BC patients. However, resistance inevitably occurs due to multifaceted factors and thus impedes therapeutic efficacy. Here, we highlight the mechanisms responsible for treatment resistance to each BC therapeutic strategy in the perspective of lncRNAs.

### LncRNAs in the development of endocrine resistance

The underlying mechanism of most BC risk factors (e.g. menstrual factors such as early menarche, late menopause and short menstrual cycles 53, reproductive factors such as late pregnancies 54) is the overexposure of mammary epithelium to ovarian hormones, especially estrogens and progesterone 55. This suggests that aberrant female hormones may be the primary stimulus for uncontrolled breast cell proliferation. In agreement, around 75% of BC patients are diagnosed as HER2-negative luminal subtypes (with positive expression of hormone receptors), suggesting that the ER signaling pathway driven by estrogen is a major oncogenic pathway of most BC 56. For the treatment of these luminal BC patients, endocrine therapies including selective estrogen receptor modulators (SERMs), selective estrogen receptor degraders (SERDs) and aromatase inhibitors are highly effective through the disruption of receptor binding or estrogen deprivation (**Figure 1**). SERMs such as the first-line endocrine agent tamoxifen can bind ER to antagonize the activity of estrogen, leading to transcriptional repression of ER target genes 57, 58. SERDs are clinically effective by not only antagonizing ERs but also degrading them, as exampled by the only FDA-approved drug fulvestrant 59. Aromatase inhibitors such as letrozole, anastrozole, and exemestane block the biosynthesis of estrogens from adrenal steroids 60. Unfortunately, most of the patients treated with these endocrine therapies finally develop treatment resistance accompanied by poor prognosis. Therefore, a deeper understanding of the potential mechanisms is urgently needed for exploiting new effective therapeutic strategies.

**Figure 2:**
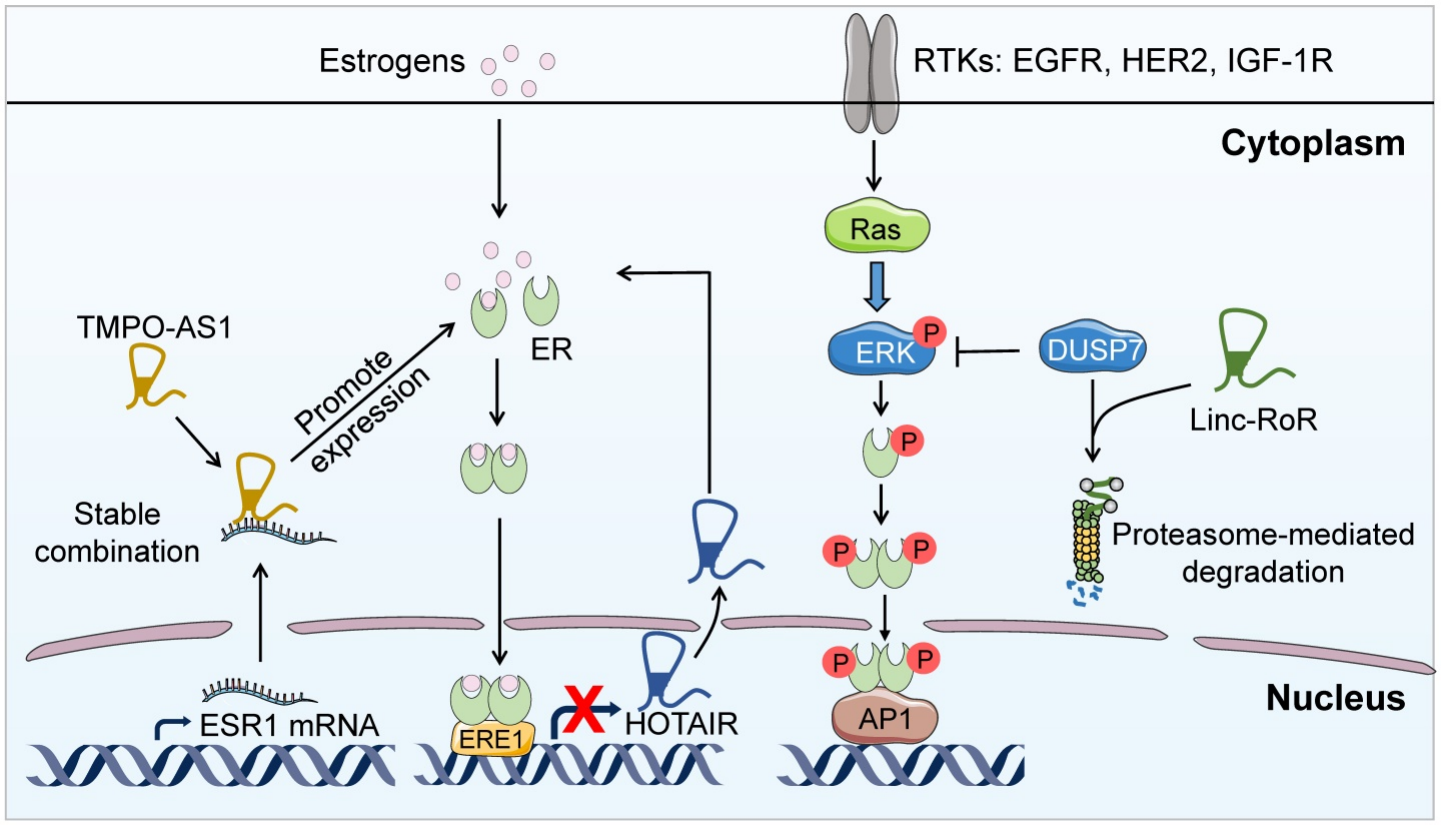
** LncRNAs-mediated endocrine therapy resistance.** LncRNA TMPO-AS1 directly interacts and stabilizes the mRNA of the ERα encoding gene *ESR1*, leading to the hyper-proliferation of ER^+^ BC and endocrine resistance. In addition, linc-RoR promotes the degradation of the ERK-specific phosphatase DUSP7 thus enhancing ERK phosphorylation. The upregulation of MAPK/ERK pathway activates ER signaling independent of estrogen, resulting in intrinsic resistance to endocrine therapy. Furthermore, HOTAIR is transcriptionally suppressed by ER. Upon blocking ER signaling through endocrine therapy, HOTAIR is upregulated and promotes the expression of ER at the protein level, leaing to enhanced transcriptional activity of ER and accelerated endocrine resistance.

BC cells develop multiple mechanisms to promote intrinsic and acquired resistance to endocrine therapy. Ligand-independent transactivation of ER is one of the major mechanisms responsible for *de novo* endocrine therapeutic resistance, in which certain lncRNAs play essential roles. It has been long described that ERK-induced ER phosphorylation elicits the estrogen-independent activation of ER signaling 22, 23. Recently, it has been reported that linc-RoR is able to activate the MAPK/ERK signaling pathway through the regulation of the ERK-specific phosphatase DUSP7 in ER^+^ BC, which bypasses the ER signaling pathway thus facilitating the development of intrinsic resistance to endocrine therapy 25 (**Figure 2**). The above study demonstrates an alternative lncRNA-dependent pathway to activate ER signaling, further illustrating the fundamental role of lncRNAs in BC endocrine resistance. Disrupting linc-RoR-mediated ER activation may help reverse endocrine resistance.

In addition, BC cells can survive endocrine therapeutic pressures through deregulation of the ER signaling components. Accumulating evidence indicates the involvement of lncRNAs in this mechanism. For example, lncRNA thymopoietin antisense transcript (TMPO-AS1) could interact with and stabilize the mRNA of the ERα encoding gene *ESR1*, leading to the hyper-proliferation of ER^+^ BC and possible endocrine resistance 61 (**Figure 2**). Besides, some lncRNAs are direct targets of ER and can possibly accelerate endocrine resistance resulting from ER signaling blockade. For instance, HOTAIR 11 and LINP1 62 are transcriptionally suppressed by ER and therefore upregulated upon blocking ER signaling following endocrine therapy. Paradoxically, upregulated HOTAIR in turn promotes the expression of ER at the protein level and facilitates its transcriptional activity 11 (**Figure 2**). However, LINP1 overexpression downregulates the protein level of ER and diminishes the estrogen response to mediate anti-estrogen resistance 62. The study of HOTAIR in endocrine resistance elucidates a positive feedback loop between ER and HOTAIR. Disrupting this loop may exhibit promising efficacy for the reversal of endocrine resistance. Moreover, DSCAM-AS1 has been found to be transcriptionally regulated by ER and confer tamoxifen resistance in BC by interacting with hnRNPL, but the detailed mechanism remaining to be determined 63.

Remarkably, a large number of lncRNAs regulate endocrine resistance in BC through the lncRNA-miRNA-mRNA axis, involving GAS5 64, urothelial carcinomaassociated 1 (UCA1) 65, CYTOR 66, DSCAM‐AS1 67 and lncRNA-ROR 68. This indicates that lncRNAs as ceRNAs have profound implication in controlling endocrine response of BC. In addition, a newly published study has demonstrated that lncRNA HOTAIRM1 promotes acquired tamoxifen resistance in BC by interacting with EZH2, thus preventing the PRC2 complex-mediated H3K27me3 of the putative HOXA1 promoter 69. This study indicates that HOTAIRM1 is a promising therapeutic target for BC patients with tamoxifen resistance.

### LncRNAs in targeted therapy resistance

BC patients with HER2 overexpression or amplification have benefited significantly from HER2-tergeted therapeutics since the first anti-HER2 monoclonal antibody trastuzumab was developed in 1990 70. Currently, the first-line treatment for HER2-positive metastatic BC is trastuzumab and pertuzumab plus docetaxel 71, while the antibody-drug conjugate trastuzumab emtansine (T-DM1) is used as second-line therapy 72 (**Figure 1**). Broader HER2-targeted therapies, especially small molecules targeting HER2 such as lapatinib 73 and neratinib 74, have also been approved for the treatment of HER2^+^ BC patients. Anti-HER2 targeted therapies have appreciably prolonged overall survival of BC patients. In addition, targeted therapies beyond HER2, including inhibitors of PI3K/AKT/mTOR pathway, cyclin-dependent kinases (CDKs) and poly (ADP-ribose) polymerase (PARP), have been applied in clinical practice to prolong the survival for patients diagnosed with BC (**Figure 1**). These targeted therapies have exhibited superior efficacy in clinical trials and have greatly benefited BC patients. However, the effectiveness of targeted therapies has been largely restricted by high rates of resistance, and lncRNAs are emerging as pivotal regulators to mediate therapeutic tolerance.

#### Therapies targeting HER2

**Figure 3:**
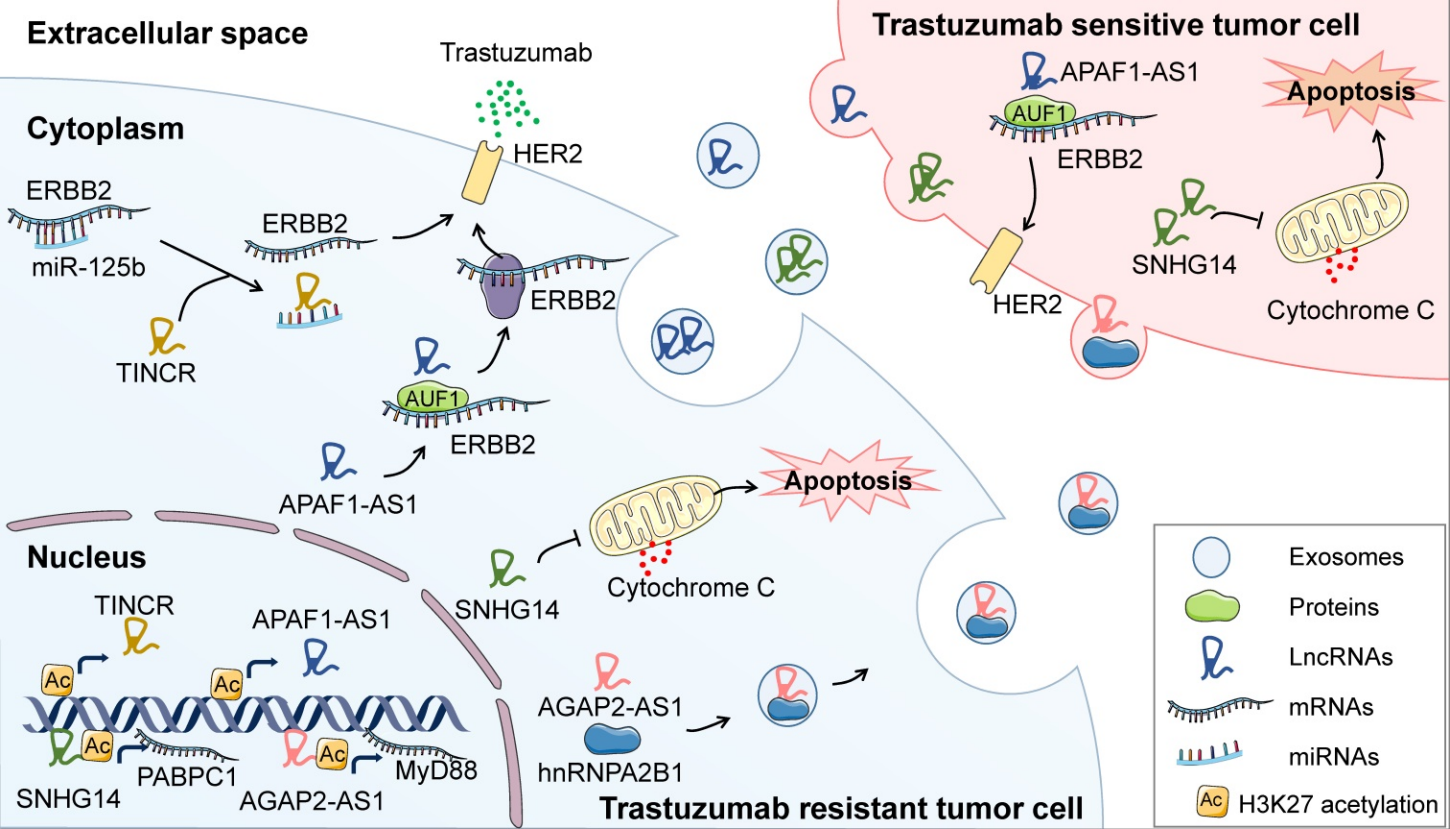
** Regulatory function of lncRNAs in BC resistance to anti-HER2 targeted therapy.** Under trastuzumab exposure, TINCR is upregulated by H3K27 acetylation and induces trastuzumab resistance through sponging miR-125b and releasing HER2 in BC cells. Moreover, AFAP1-AS1 can be upregulated through H3K27 acetylation at its promoter region and guides AUF1 to bind to HER2 mRNA, leading to enhanced translation of HER2. AGAP2-AS1 increases H3K27 acetylation at the promoter region of MyD88, resulting in the activation of NF-κB signaling pathway and therapeutic resistance to trastuzumab. Moreover, another lncRNA, known as SNHG14, could inhibit trastuzumab-induced apoptosis through upregulating Bcl2. Intriguingly, several lncRNAs have been reported to confer trastuzumab resistance in surrounding cells through being engulfed in exosomes and incorporated by neighbor cells.

LncRNAs have attracted increasing attention as pivotal regulators of trastuzumab resistance in BC. Some studies have revealed that lncRNAs which confer acquired trastuzumab resistance in BC cells can be incorporated into exosomes thus disseminating the resistance to surrounding cells (**Figure 3**). For example, in trastuzumab resistant BC cells, lncRNA AFAP1-AS1 is upregulated through the H3K27 acetylation at its promoter region and guides AUF1, which improves the translation of target mRNA, to bind to HER2 mRNA thus enhancing HER2 translation and trastuzumab resistance. Strikingly, AFAP1-AS1 in trastuzumab resistant cells can be packaged into exosomes and promote resistance in recipient cells 75. In addition, lncRNA AGAP2-AS1 76 and SNHG14 77, 78 can also facilitate trastuzumab tolerance of BC cells through exosome-mediated dissemination. These findings reveal that exosomes play fundamental roles in lncRNAs-mediated BC resistance to targeted therapies. Further, exosomal lncRNAs have also been widely documented to modulate cancer therapeutic resistance in other types of tumors 79. Therefore, in addition to target lncRNA, blocking the packaging or secretion of exosomes may become a promising strategy for attenuating therapeutic resistance.

According to a recent study, another lncRNA, TINCR, can be activated by CREB-binding protein (CBP)-mediated H3K27 acetylation, leading to trastuzumab resistance in BC 21. Mechanistically, TINCR acts as a sponge for miR-125b, thus releasing HER2 to compromise the anti-tumor effect of trastuzumab 21. Perhaps the most common functional mechanism of lncRNAs is remodeling chromatin structure to regulate gene expression, through which lncRNAs may enhance the resistance to trastuzumab treatment. For example, lncRNA AGAP2-AS1 induced by the transcription factor SP1 binds to CBP and increases H3K27 acetylation at the promoter region of MyD88, resulting in the activation of the NF-κB signaling pathway and resistance to trastuzumab 80. Other lncRNAs, as exampled by H19 81, UCA1 82 and GAS5 83 have been documented to be closely related to trastuzumab resistance; however, the mechanisms are as yet to be determined. In spite of increased understanding of lncRNAs-regulated trastuzumab resistance, the involvement of lncRNAs in resistance to pertuzumab and other small molecules targeting HER2 needs further exploration.

#### Therapies targeting PI3K/AKT/mTOR pathway

The PI3K/AKT/mTOR pathway is commonly hyperactivated in BC due to frequent somatic PIK3CA mutations and HER2-triggered oncogenic signaling. Some PI3K inhibitors have been launched for clinical use in BC treatment (**Figure 1**). For example, the novel and specific PI3Kα inhibitor NVP-BYL719 is now under active preclinical and clinical studies for the treatment of BC patients with PIK3CA mutations and/or HER2 amplification 84. In particular, alpelisib, which is indicated for use in combination with fulvestrant in ER^+^, HER2^-^ BC patients, is the first PI3K inhibitor that has been approved by the FDA 85. The mTOR inhibitor everolimus increases PFS by more than twofold in ER^+^, HER2^-^ advanced BC patients after failure of treatment with nonsteroidal aromatase inhibitors 2. In addition, a phase-II clinical trial has demonstrated that the pan-AKT inhibitor MK2206 could increase pathologic complete response rates when combined with standard neoadjuvant therapy in ER^-^/PR^-^ and HER2^+^ BC 86. The AKT inhibitors capivasertib and ipatasertib in combination with first-line paclitaxel therapy significantly prolong the PFS of TNBC patients, especially those with PIK3CA/AKT1/PTEN-alterations 87, 88. However, the PI3K/AKT/mTOR axis may be reactivated by compensatory signaling pathways dependent on lncRNAs, resulting in resistance to targeted therapies.

**Figure 4:**
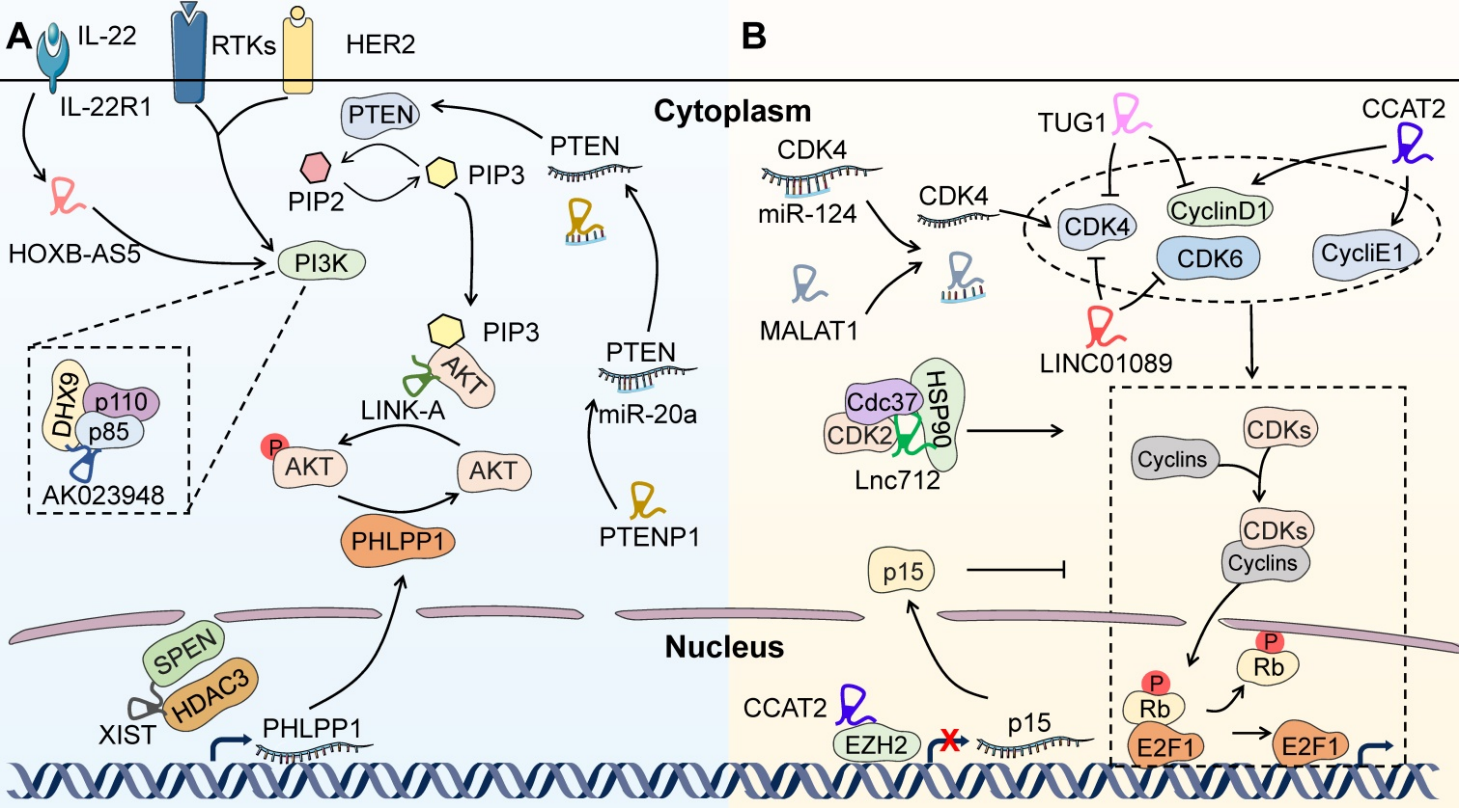
** LncRNAs-regulated resistance to targeted therapies beyond HER2.** LncRNAs are emerging as pivotal regulators to mediate therapeutic tolerance to targeted therapies beyond HER2, including inhibitors of PI3K/AKT/mTOR pathway, CDKs and PARP. (A) LncRNAs-mediated resistance to therapies targeting PI3K/AKT/mTOR pathway. LINK-A directly binds to AKT and PIP3 to facilitate the enzymatic activation of AKT, leading to BC resistance to MK2206. AK023948 interacts with DHX9 and p85 to positively regulate AKT. In addition, IL-22-induced HOXB-AS5 expression could activate PI3K. Conversely, PTENP1 sponges miR-20a to release PTEN and negatively regulates PI3K/AKT/mTOR pathway. Furthermore, XIST can sequester HDAC3 to enhance the transcription of PHLPP1 and dephosphorylation of AKT. (B) LncRNAs-mediated resistance to therapies targeting CDKs. Lnc712 activates CDK2 through directly interacting with HSP90 and forming a complex of Lnc712/HSP90/Cdc37. In addition, MALAT1, TUG1, CCAT2 and LINC01089 are associated with the altered function of CDKs and Cyclins, which may be required for the drug resistance to CDK inhibitors in BC cells.

A recent study has shown that LINK-A directly binds to AKT and PIP3 to enhance the interaction between them, thus facilitating the enzymatic activation of AKT. LINK-A-induced hyperactivation of AKT is responsible for BC resistance to MK2206, which raises a hurdle to AKT targeted therapy in BC patients 89 (**Figure 4A**). Therefore, LINK-A may be a promising biomarker and therapeutic target for predicting AKT inhibitor efficacy and reversing treatment tolerance. Besides, a CRISPR/Cas9-based synergistic activation mediator (SAM) system has been developed and identified lncRNA AK023948 as a positive AKT regulator in BC through interacting with ATP-dependent RNA helicase A (RHA/DHX9) and p85 to sustain the stability of p85 90 (**Figure 4A**). Therefore, AK023948-regulated AKT activation may facilitate resistance to AKT-targeted therapies in BC patients. Recently, Zhou *et al*. have documented that Linc-ROR decreases BC cell sensitivity to mTOR inhibitor rapamycin by sponging miR-194-3p and releasing methyl CpG-binding protein 2 (MECP2) 91. In spite of the puzzling mechanism, this study proves a direct link between lncRNA and BC resistance to therapies targeting PI3K/AKT/mTOR pathways, again suggesting that lncRNAs are promising therapeutic targets to overcome BC resistance. In addition, it has been reported that IL-22 and the lncRNA HOXB-AS5 could synergistically activate the PI3K/AKT/mTOR pathway 15 (**Figure 4A**). Remarkably, IL-22 and HOXB-AS5 are detectable in the serum of BC patients, both of which are upregulated and closely related to the clinical stage of BC. Therefore, HOXB-AS5 represents an ideal biomarker to predict therapeutic response to PI3K/AKT/mTOR targeted therapy, which still requires further validation before entering the clinic 15. Many other lncRNAs have also been reported to be capable of regulating PI3K/AKT/mTOR pathway, such as PTENP1 92, 93, MALAT1 94 and Xist 95, indicating the possible involvement of these lncRNAs in regulating resistance to the targeted therapies in BC (**Figure 4A**). However, the underlying mechanism remains largely unknown and requires further elucidation.

#### Therapies targeting CDKs

Preclinical and clinical studies have demonstrated the synergistic effect of CDKs inhibitors with anti-estrogen agents, probably due to the special dependence of ER^+^ BC cells on cyclin D1 and estrogen-mediated activation of CDKs 96. CDK4/6 inhibitors such as palbociclib 97, ribociclib 98 and abemaciclib 99 combined with endocrine agents have exhibited significant PFS benefit and are approved by the FDA as the standard treatment for ER^+^, HER2^-^ advanced BC patients (**Figure 1**). Furthermore, a potent dual inhibitor of CDK12/CDK13 has been developed and demonstrated to provoke TNBC cell death by suppressing the pivotal DNA damage response genes and triggering lethal accumulation of DNA damage 100. This study raises the exciting possibility of developing targeted therapies for TNBC, but still needs further clinical investigation. As with all the other therapeutic strategies, the emergence of resistance to CDK inhibitors is a major clinical obstacle.

The most common mechanism underlying resistance to CDK4/6 inhibitors is cell cycle alterations 101, with some clues implying the participation of lncRNAs (**Figure 4B**). It has been shown that the responsiveness of CDK4/6 inhibitor palbociclib could be restricted by elevated CDK2 expression or activity, suggesting that redundant CDK functions may predict treatment failure for CDK inhibitors 102, 103. A newly identified lncRNA, Lnc712, could activate CDK2 by directly interacting with heat-shock protein 90 (HSP90) and forming a complex of Lnc712/HSP90/cell division cycle 37 (Cdc37) in BC 16. These findings indicate that Lnc712 may enhance resistance to palbociclib and become a promising biomarker for the prediction of drug response in BC. Similarly, lncRNAs associated with other CDKs, including MALAT1 104, TUG1 105, CCAT2 106 and LINC01089 107, may also mediate resistance to CDK inhibitors. Overall, the direct nexus between lncRNAs and CDK inhibitors is not yet well established. Further investigation focused on the regulation of CDK inhibitor responsiveness by lncRNAs may help to understand the mechanisms underlying treatment resistance.

#### Therapies targeting PARP

PARP is an enzyme able to initiate single-strand DNA break repair by synthesizing a polymeric adenosine diphosphate ribose (PAR) chain and recruiting critical DNA-repairing enzymes. PARP inhibition leads to DNA double-strand breakage, which is normally repaired by homologous recombination (HR) dependent on BRCA1 and BRCA2 108. Since BRCA mutation predisposes to certain cancers as exampled by BC, it can be anticipated that BRCA-mutated BC will be sensitive to PARP inhibitors. Supporting this, two PARP inhibitors, olaparib (Lynparza) and talazoparib (Talzenna), received FDA approval for the treatment of germline BRCA-mutated, HER2^-^ locally advanced or metastatic BC in 2018.

Nevertheless, the benefit of PARP inhibitors has been shown to be heavily compromised by drug resistance. The main mechanisms of PARP inhibitor resistance identified to date include disrupting cellular drug availability, affecting (de)PARylation enzymes, reactivating HR and restoring replication fork stability 109. Emerging evidence shows the intimate correlation between lncRNAs and DNA damage repair, strongly supportive of lncRNAs involvement in PARP inhibitor resistance. For example, ataxia-telangiectasia mutated (ATM)-mediated DNA damage response has been found to induce the expression of lncRNA-JADE. lncRNA-JADE directly binds to BRCA1 and transcriptionally activates Jade1, a critical element in human acetylase binding to ORC1 (HBO1) histone acetylation complex. The Jade1 activation mediated by lncRNA-JADE promotes global histone H4 acetylation and increases transcription of DNA damage repair-related genes 110. Linc00261 has been demonstrated to be epigenetically regulated by FOXA2 and induce phosphorylation and activation of DNA damage machinery 111. Other lncRNAs, such as mitotically-associated long noncoding RNA (MANCR) 112 and transcribed in the opposite direction of RAD51 (TODRA) 113, are revealed to maintain genomic stability and induce HR. Given the capability of these lncRNAs to enhance DNA damage repair and sustain genomic stability, it can be assumed that lncRNAs may play an essential role in PARP inhibitor resistance.

Moreover, in consideration of the key role of the tumor suppressor p53 in maintaining genome stability 114, it is conceivable that an enigmatic nexus may exist between p53 status and PARP inhibitor response. Recently, some p53-responsive lncRNAs have been reported to play essential roles in BC, indicating their potential involvement in PARP inhibitor resistance. For instance, the lncRNA GUARDIN induced by p53 plays an essential role in the maintenance of genome integrity by sustaining the stability of telomeric repeat-binding factor 2 (TRF2) and BRCA1 115. The correlation between GUARDIN and p53 as well as the competence of GUARDIN to maintain BRCA1 expression imply that GUARDIN may confer intrinsic resistance to PARP inhibitor. Another lncRNA in the nonhomologous end joining (NHEJ) pathway (LINP1) is also regulated by p53 and overexpressed in TNBC 116. LINP1 can facilitate DNA double-strand breaks repair, suggesting its potential to counteract PARP inhibitor-induced DNA damage repair vulnerability 116. An exciting recent study has revealed that downregulated lncRNA PHACTR2-AS1 in BC is associated with tumor development and poor prognosis. Aberrant activation of EZH2 targets and downregulates PHACTR2-AS1, which triggers H3K9 methylation-mediated silencing of ribosome DNA genes, thus inducing genome instability 117. This finding further implicates the involvement of lncRNAs in DNA damage signaling. However, the direct correlation between lncRNAs and PARP inhibitor resistance remains to be established.

### LncRNAs in BC chemoresistance

Chemotherapy is beneficial to the treatment of almost every subtype of BC. Currently, anthracylines and taxanes are standard chemotherapy for early stage BC 1 (**Figure 1**). Anthracylines such as doxorubicin exert their cytotoxic functions via pleiotropic mechanisms including macromolecular biosynthesis inhibition, free radical production, and DNA damage induced by histone eviction from open chromatin 118, 119. Taxanes (e.g. paclitaxel and docetaxel) can bind and stabilize microtubules to prevent depolymerization and block mitosis progression 120, 121. For HER2^+^ metastatic BC, as mentioned above, trastuzumab in combination with taxane chemotherapy has been found to improve overall survival (OS) and has been the first-line standard treatment since 2001 122. Due to the lack of specific biomarkers in TNBC, targeted therapies have rarely met the need to improve clinical outcomes. Therefore, chemotherapy is always recommended as the standard of care for TNBC patients.

Currently, dysregulated lncRNAs have been widely documented to play dual roles in BC chemoresistance. Among all the studies available to date, most of the lncRNAs enhance chemoresistance in BC by acting as ceRNAs to sponge miRNAs, especially through targeting the ATP-binding cassette (ABC) transporter superfamily. The ABC transporter superfamily as drug efflux pumps have been known to mediate multidrug resistance (MDR) in multiple cancers 123. For example, lncRNA ferritin heavy chain 1 pseudogene 3 (FTH1P3) and linc00518 respectively sponge miR-206 and miR-199a, which share complementary binding sites with ABCB1 and MRP1 mRNA, thus mediating MDR (e.g. paclitaxel, doxorubicin and vincristine) in BC 124, 125. Besides, dysregulated NONHSAT101069 is reported to sponge miR-129-5p and release Twist1 to confer resistance to the anthracycline genotoxic drug epirubicin in BC 126, and CASC2 enhances paclitaxel resistance through regulation of the miR-18a-5p-CDK19 axis 127. On the basis of several independent studies, NEAT1 confers resistance to paclitaxel, cisplatin and 5-fluorouracil in BC cells through miR-129/ZEB2 and miR-211/HMGA2 pathways 12, 13. Collectively, the mechanism whereby lncRNAs act as ceRNAs to release specific mRNAs is widely seen in BC chemotherapy resistance. Specifically targeting the lncRNA-miRNA-mRNA axis may benefit BC patients resistant to chemotherapy.

In addition to acting as ceRNAs, lncRNAs can exert their function of mediating BC chemoresistance through multiple mechanisms. Of note, a recent study has reported a special mechanism of the antisense lncRNA MAPT-AS1 to mediate chemoresistance in BC 128. In detail, MAPT-AS1 contributes to paclitaxel resistance in ER^-^ BC cells through the formation of RNA duplex with its natural comparable sense transcripts MAPT. The RNA duplex may alter the spatial structure and increase the stability of MAPT mRNA, which has been demonstrated to be involved in BC chemoresistance 128. LncRNA NONHSAT141924 enhances BC resistance to paclitaxel through the p-CREB/Bcl-2 apoptosis pathway 129. Intriguingly, H19 can induce doxorubicin resistance in BC, and be engulfed into exosomes to disseminate the resistance to surrounding sensitive cells 130, 131. Thus, targeting H19 in BC cells as well as blocking its incorporation into exosomes may exhibit superior efficacy in reversing BC chemoresistance.

Compared with lncRNAs which promote chemoresistance in BC, fewer lncRNAs have been found to suppress chemoresistance in BC and the underlying mechanisms still remain elusive. For instance, LINC00968 targets and silences WNT2 and the downstream β-catenin signaling pathway, thus sensitizing BC cells to chemotherapeutics 132. LncRNA GAS5 suppresses adriamycin resistance by competing with miR-221-3p for DKK2 release, which downregulates the Wnt/β-catenin signaling pathway 133. Besides, AC073284.4 attenuates paclitaxel resistance via inhibition of miR-18b-5p/dedicator of cytokinesis protein 4 (DOCK4) axis 134. Taken together, lncRNAs are extensively involved in BC chemoresistance. Modulating the expression of chemoresistance-related lncRNAs is a feasible strategy for the reversal of resistance.

### LncRNAs in BC immunotherapy resistance

Currently, there is a growing enthusiasm for the application of immunotherapy in BC treatment, with the immune checkpoint blockade playing the leading role 135 (**Figure 1**). Agents causing immune checkpoint blockade are under active clinical investigation, either used alone or in combination with other therapeutics. For example, some chemotherapeutics and targeted agents appear promising in combination with immune checkpoint inhibitors because of their competence in T-cell priming and immunity activation 135. Of note, in 2019 the immune checkpoint blockade agent atezolizumab (Tecentriq) was approved to be paired with cytotoxic chemotherapeutic nab-paclitaxel (Abraxane) for the first-line treatment of PD-L1^+^ unresectable locally advanced or metastatic TNBC. This initial approval of an immunotherapeutic marks a milestone in BC treatment, especially in TNBC, since prior to that cytotoxic chemotherapeutics were the only treatment option for these patients.

**Figure 5:**
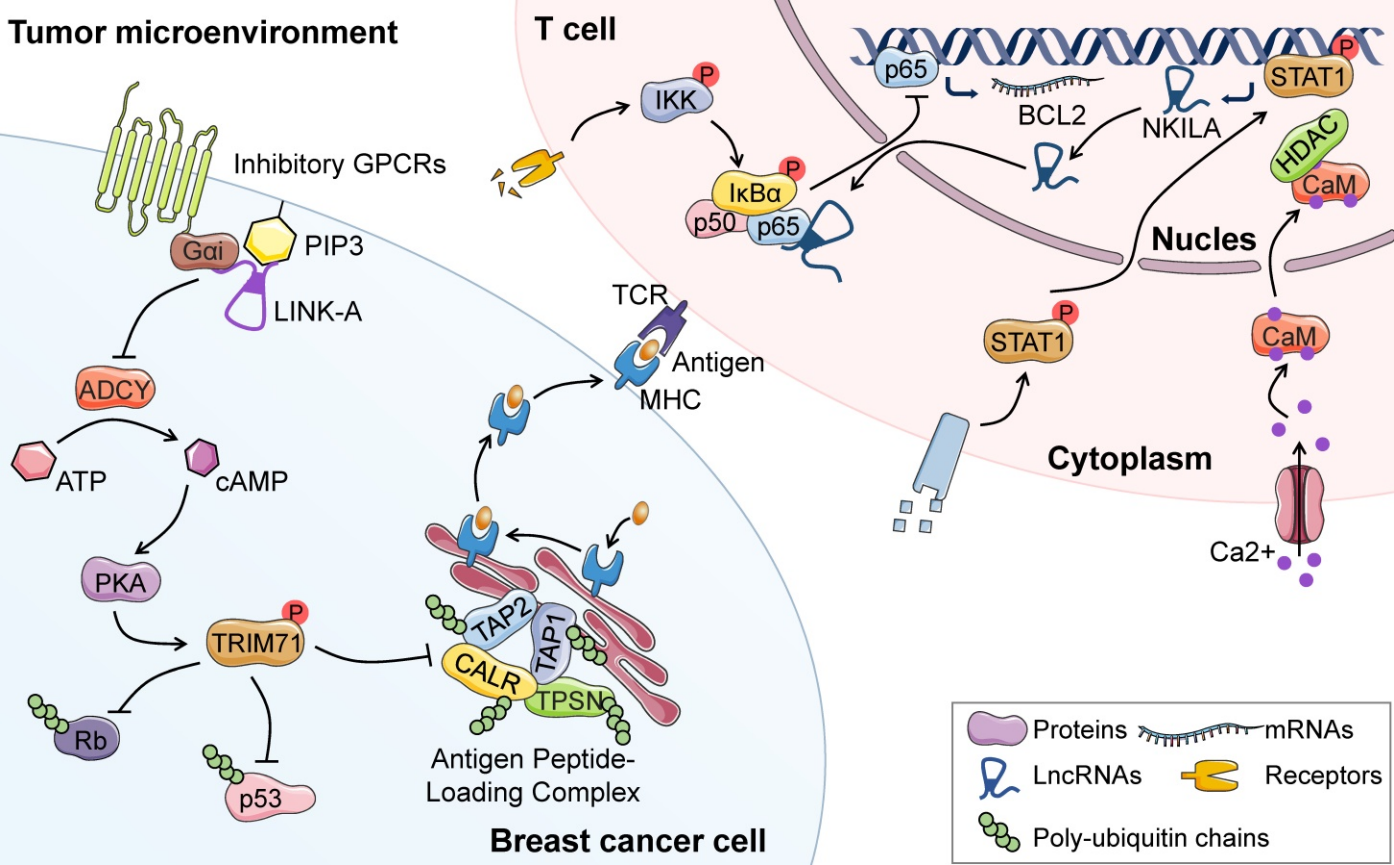
** LncRNAs-regulated resistance to immunotherapy.** In BC cells, LINK-A promotes the crosstalk between PIP3 and inhibitory GPCR pathways, leading to decreased cAMP and PKA-mediated phosphorylation of TRIM71. Decreased phosphorylation of TRIM71 enhances the degradation of PLC, Rb and p53, resulting in downregulation of antigenicity and intrinsic tumor resistance to immunotherapy. In stimulated T cells, calcium influx activates calmodulin, thereby removing HDAC and enhancing STAT1-mediated transcription of NKILA. NKILA directly binds to p65 and prevents its nuclear translocation as well as following transcription of anti-apoptotic genes. Thus, NKILA facilitates T cell vulnerability to AICD, resulting in immune evasion and cancer progression of BC.

In spite of the encouraging progress in BC immunotherapy, cancer cells can develop variable mechanisms to escape immunosurveillance and generate intrinsic unresponsiveness to immunotherapeutics. One of the immune-resistant mechanisms involves reducing antigenicity that avoids detection by antitumor lymphocytes. For instance, TNBC exhibits resistance to programmed cell death protein-1(PD-1) blockade through downregulation of PLC, which enhances antigen presentation to the cell surface 136. More intriguingly, the lncRNA LINK-A plays a key role in this process by promoting the degradation of PLC. In agreement with this, treatment with LINK-A LNAs stabilizes PLC components and sensitizes mammary gland tumors to immune checkpoint inhibitors 17 (**Figure 5**). This study gives an example of lncRNA-dependent antigenicity reduction and intrinsic immunotherapy resistance, which underscores the significance of lncRNAs as potential therapeutic targets and novel biomarkers to predict immunotherapy responses of BC patients.

In addition to the downregulation of antigenicity, transformed cells take advantage of AICD of T lymphocytes to escape immunological elimination, in which the lncRNAs play an important role (**Figure 5**). For example, a recent study has demonstrated that an NF-κB-interacting lncRNA, NKILA, facilitates T cell vulnerability to AICD through inhibition of NF-κB activity, resulting in BC immune evasion and cancer progression. Moreover, knockdown of NKILA in tumor-specific CTLs significantly inhibits the growth of BC patient-derived xenografts due to increased CTL infiltration 52. Thus, modulating the expression of oncogenic lncRNAs such as NKILA in CTL may represent a novel immunotherapeutic strategy. Moreover, the expression levels of XIST and TSIX are related to PD-L1 in the tissues and body fluids of BC patients with diverse molecular subtypes, indicating the role of the two lncRNAs as predictive biomarkers for immunotherapy in BC patients 137. Emerging studies have shed light on the mechanism by which XIST attenuates immune suppression. For instance, XIST depletion enhances the secretion of exosomal miRNA-503 to activate M1-M2 conversion of microglia, which is essential for maintaining the suppressive immune microenvironment and inhibiting T cell proliferation 138. In addition to those lncRNAs mentioned above, multiple lncRNAs have been found to be associated with immune system regulation, including RP4-583P15.10 139, T-cell leukemia/lymphoma 6 (TCL6) 140, growth hormone secretagogue receptor opposite strand (GHSROS) 141, and linc00152 142. These findings demonstrate that lncRNAs may act as promising predictive biomarkers and therapeutic targets for BC immunotherapy.

## Clinical implications of lncRNAs in BC therapy

Early stage BC is considered to be potentially curable while the treatment for metastatic or late BC is still challenging, suggesting that early and precise diagnosis is extremely important for BC patients. Currently, two-view mammography is the sole screening method recommended for early detection of BC 6. However, the high frequency of false positives or negatives limits the reliability of this clinically used screening tool and may impact on survival or impair life quality of patients 143. Interestingly, a deep learning-based model can outperform radiologists in identifying BC from mammograms, but the clinical benefits are not yet fully established 144. Many state-of-the-art imaging methods, including abbreviated MRI 6 and digital breast tomosynthesis (DBT) 145, are being developed to expand the sensitivity profile of early stage BC detection. However, the enormous cost, sensitivity and specificity of these screening methods remain to be optimized.

Although ER, PR, and HER2 provide insight into BC molecular classification and clinical management, novel biomarkers for BC subtyping are urgently needed to facilitate individualized treatment due to the high intertumor heterogeneity in BC patients. In addition to the lack of effective biomarkers for early detection and molecular subtyping, resistance against standard therapies has also increased the mortality of BC as discussed above. Therefore, sensitive biomarkers and effective therapeutic targets are urgently required to improve the clinical outcomes of BC patients.

### LncRNAs as biomarkers to predict therapeutic response

Mining novel biomarkers for BC management is extremely important for improving clinical outcomes. Some lncRNAs have been found to act as essential regulators in BC, indicating their potential to serve as biomarkers for early diagnosis and molecular subtyping. For instance, Xu *et al.* has reported that lncRNA RP11-445H22.4 in circulating serum is a sensitive and specific diagnostic biomarker for BC 146. Liu *et al.* have developed a novel classification system integrating lncRNA and mRNA expression profiles for TNBC and demonstrated that subtype-specific lncRNAs could act as potential biomarkers for BC classification 147. Another recent study has demonstrated that the overexpression of HOTAIR is intimately associated with a luminal androgen receptor (LAR) subtype of TNBC, which is characterized by AR expression 148. These findings indicate the prognostic significance of lncRNAs in BC and raise the possibility of establishing novel detection strategies for BC patients.

In addition, many lncRNAs have been found to be capable of predicting therapeutic response. High expression of HOTAIR and its regulator FOXM1 can help identify endocrine therapy non-responders among ER^+^ BC patients, illustrating the role of lncRNAs as predictive biomarkers in BC therapy 149. In addition, the histone deacetylase inhibitor abexinostat has been demonstrated to induce BCSCs differentiation and reduce BCSC population *in vivo*. XIST functions as a predictive biomarker to distinguish BC patients sensitive to this differentiation therapy 150. XIST^low^ BC cells can be specifically eliminated by a FDA-approved chemotherapy medication fludarabine 138. Further, multigene signatures incorporating lncRNAs have been developed and demonstrated to be capable of predicting taxane responsiveness, tumor recurrence and clinical outcome in patients diagnosed with TNBC 151, 152. The functionally polymorphic lncRNA MIR2052HG can influence the risk of tumor recurrence in BC patients treated with aromatase inhibitors 153. These studies reveal that lncRNAs can serve as active biomarkers for therapeutic response prediction, but need further validation in clinical practice. This would be beneficial for personalized/precision medicine.

Interestingly, lncRNAs, including Prostate Cancer gene 3 (PCA3), MALAT1, H19, TINCR and CCAT2, are emerging as circulating biomarkers for early stage cancer detection 154, 155. A recent study has reported that extracellular RNAs (exRNAs) from a single droplet (5-7 µL) of serum in a liquid biopsy are capable of reflecting human physiological and disease states using SILVER-seq (Small Input Liquid Volume Extracellular RNA Sequencing). More intriguingly, lncRNAs are detectable in this system, and donors with or without BC display significant differences 156. These findings indicate that lncRNAs can act as sensitive biomarkers in BC non-invasive liquid biopsy, but again this needs further clinical validation. Unfortunately, the BC patient cohorts used in the above studies are relatively small, and larger studies are required to validate the clinical value of these lncRNAs as biomarkers.

### LncRNAs as promising BC therapeutic targets

Notwithstanding the several therapeutic strategies available for BC patients, overcoming treatment tolerance remains a major challenge for improving clinical outcome. Theoretically, the regulatory roles of lncRNAs in BC therapeutic resistance confer them potential to be targeted for reversing treatment tolerance. Furthermore, in practice, the development of lncRNA-targeting technologies makes it possible to translate lncRNA-based therapies to the clinic. To date, the most general approaches for suppressing lncRNA involve antisense oligonucleotides (ASOs) and small interfering RNAs (siRNAs) 157. ASOs refer to synthetic single-stranded oligonucleotides which are complementary to target lncRNAs and can form a DNA/RNA heteroduplex which can be cleaved by RNase H. siRNAs are double-stranded RNAs, the guide strand of which can be loaded into argonaute 2 (AGO2) and then form a RNA-induced silencing complex (RISC) to target lncRNA for degradation. ASOs predominantly target lncRNAs residing in the nucleus while cytoplasmic lncRNAs are more sensitive to siRNAs 158.

Both of these two methods have been applied to silence lncRNAs and have shown considerable efficacy against BC in preclinical studies. Genetic knockout or knockdown of MALAT1 159, LINC02273 160 and LINC00673 161 using ASOs has exhibited superior efficacy in attenuating BC growth and metastasis* in vivo*. siRNAs targeting BC-related lncRNAs such as HOTAIR have also been demonstrated to inhibit BC growth and invasion 162. Remarkably, silence of lnc-BM with siRNAs encapsulated within nanoparticles has shown considerable efficacy against brain metastasis in BC 163, indicating that ASOs or siRNAs formulated into suitable drug delivery vehicles (e.g. liposomes, nanoparticles and viruses) may accelerate the clinical application of lncRNAs-based treatment strategies.

LNAs are well accepted as a novel class of RNA used in therapeutics. The ribose moiety of the RNA nucleotide is locked by an extra oxymethylene bridge linking the C (2') and C (4') to facilitate base stacking and enhance hybridization properties 164, 165. The unique characteristics of LNAs, including high binding affinity, high stability and improved mismatch discrimination, make them emerging approaches for BC treatment 166. Therapeutic delivery of LNAs targeting LINK-A 17 and BCAR4 167, 168 has been proven to sensitize BC to immune checkpoint inhibitors and attenuate BC growth and metastasis *in vivo*, respectively. Preclinical studies on lncRNA-based BC therapy have made substantial progress and offered an opportunity to reverse BC therapeutic resistance. However, these studies are still preliminary and great challenges lie ahead for translating these treatment methods into the clinic, requiring efforts in oligonucleotide chemistry and development of appropriate delivery systems.

## Conclusions

In the past few decades, studies on lncRNAs in the field of oncology have drawn considerable attention of researchers, largely due to the rapid and substantial progress in high-throughput sequencing technologies. Additionally, the aberrant expression and multifaceted roles of lncRNAs in cancer have also attracted increasing scientific interests. In this review, we summarize the dysregulated lncRNAs associated with resistance to current BC therapeutic strategies and highlight the underlying mechanisms to facilitate the understanding of BC treatment tolerance.

The lncRNAs implicated in BC therapy resistance hold potential to serve as predictive biomarkers or therapeutic targets to benefit clinical management of BC patients. Firstly, lncRNAs have been widely documented as biomarkers for BC diagnosis, prognosis and therapeutic response prediction. The sensitivity and specificity have been demonstrated in several cohorts of BC patients. It therefore appears that lncRNAs are promising biomarker candidates to be translated into clinical applications. Unfortunately, the size of many clinical studies conducted to date is not large enough to verify their clinical value. Thus, larger BC patient cohorts are needed to validate the potential of these lncRNAs as clinical biomarkers.

Furthermore, the prerequisite of translating lncRNAs to the clinic as therapeutic targets is to elucidate their elaborated mechanisms of action *in vitro* and *in vivo*. However, the major impediment that lies ahead is their often obscure mode of action. Additionally, the low sequence conservation of lncRNAs poses a challenge in validating their biological functions. It has been suggested that the secondary structure of lncRNAs are evolutionarily conserved and could be considered as the major functional unit to regulate biological activities 169. However, very few studies focus on the influence of lncRNA secondary structure on their biological functions. Additionally, the same lncRNA may have multiple targets and could even exert opposite functions in different types of tumors, resulting in dramatic side effects. Lack of animal models remains another limitation in mechanistic studies on lncRNAs. Further exploration on the character of lncRNAs and more technical breakthroughs are required to address these problems. Taken together, investigation of lncRNAs in BC therapeutic resistance may further enhance our understanding of the mechanisms responsible for resistance to BC treatment. Hopefully, some of the specific lncRNAs will enter the clinic as promising prognostic biomarkers or effective therapeutic targets.

## Figures and Tables

**Table 1:** Mechanisms of BC therapeutic resistance mediated by lncRNAs

LncRNA	Therapeutic strategy	Pathway/target	Action modes	Effection	Refs
HOTAIR	Tamoxifen	ER	Promoting ligand-independent ER activities, increasing cancer stemness	Inducing	11
NEAT1	Paclitaxel , cisplatin, 5-FU	miR-129/ZEB2, miR-211/HMGA2	CeRNA, regulating apoptosis and cell cycle progression, facilitating cell growth	Inducing	12, 13
HOXB-AS5	PI3K/AKT/mTOR inhibitors	PI3K/AKT/mTOR	Promoting cell growth, migration and invasion	Inducing	15
Lnc712	CDK inhibitors	HSP90	Regulating CDK2 activation and triggering cell proliferation	Inducing	16
LINK-A	Immune checkpoint blockers	PIP3/GPCR, PLC	Reducing antigenicity to avoid detection by antitumor lymphocytes	Inducing	17
H19	Tamoxifen, fulverstrant	ER	Regulating ERα expression at the transcript and protein levels	Inducing	19
MIR2052HG	Aromatase inhibitors	EGR1, ER	Promoting *ESR1* transcription and limiting ubiquitin-mediated ERα degradation	Inducing	20
TINCR	Trastuzumab	miR-125b/ERBB2	CeRNA, regulating the expression level of HER2	Inducing	21
Linc-RoR	Tamoxifen	DUSP7, MAPK/ERK	Promoting estrogen-independent cell growth	Inducing	25
DCST1-AS1	Doxorubicin, paclitaxel	ANXA1	Unknown	Inducing	37
NKILA	Immunotherapy	NF-κB	Facilitating T cell vulnerability to AICD and decreasing CTL infiltration	Inducing	52
TMPO-AS1	Endocrine therapy	ER	Stabilizing *ESR1* mRNA through interaction with *ESR1* mRNA	Inducing	61
LINP1	Tamoxifen	ER	Attenuating the estrogen response	Inducing	62
DSCAM-AS1	Tamoxifen	hnRNPL	Unknown	Inducing	63
GAS5	Tamoxifen	miR-222/PTEN	CeRNA	Reversing	64
UCA1	Tamoxifen	miR-18a/HIF1α	CeRNA, regulating cell cycle	Inducing	65
CYTOR	Tamoxifen	miR‑125a‑5p/SRF, Hippo, MAPK	CeRNA, promoting cell survival	Inducing	66
DSCAM‐AS1	Tamoxifen	miR‐137/EPS8	CeRNA, promoting cell proliferation and suppressing apoptosis	Inducing	67
HOTAIRM1	Tamoxifen	EZH2	Preventing H3K27me3 of HOXA1	Inducing	69
AFAP1-AS1	Trastuzumab	AUF1/ERBB2	Enhancing HER2 translation, exosome-mediated dissemination	Inducing	75
AGAP2-AS1	Trastuzumab	hnRNPA2B1	Exosome-mediated dissemination	Inducing	76
SNHG14	Trastuzumab	Bcl-2/Bax, PABPC1	Inhibiting apoptosis, exosome-mediated dissemination	Inducing	77, 78
AGAP2-AS1	Trastuzumab	CBP, MyD88, NF-κB	Activating NF-κB signaling pathway, promoting cell growth	Inducing	80
LINK-A	MK2206	AKT	Facilitating the enzymatic activation of AKT	Inducing	89
AK023948	AKT inhibitors	DHX9/p85	Sustaining the stability of p85	Inducing	90
Linc-ROR	mTOR inhibitor (rapamycin)	miR-194-3p/ MECP2	CeRNA	Inducing	91
lncRNA-JADE	PARP inhibitors	BRCA1, Jade1	Increasing transcription of DNA damage repair-related genes	Inducing	110
GUARDIN	PARP inhibitors	BRCA1, TRF2	Maintaining genome integrity	Inducing	115
PHACTR2-AS1	PARP inhibitors	Ribosome DNA genes	Triggering H3K9 methylation-mediated silencing of ribosome DNA genes	Inducing	117
FTH1P3	Paclitaxel	miR-206/ABCB1	CeRNA	Inducing	124
Linc00518	Paclitaxel	miR-199a/MRP1	CeRNA	Inducing	125
NONHSAT101069	Epirubicin	miR-129-5p/Twist1	CeRNA	Inducing	126
CASC2	Paclitaxel	miR-18a-5p/CDK19	CeRNA	Inducing	127
MAPT-AS1	Paclitaxel	MAPT	Increasing the stability of MAPT mRNA	Inducing	128
NONHSAT141924	Paclitaxel	p-CREB/Bcl-2 apoptosis pathway	Unknown	Inducing	129
LINC00968	Paclitaxel, adriamycin	WNT2	Inhibiting the Wnt2/β-catenin signaling pathway	Reversing	132
GAS5	Adriamycin	miR-221-3p/DDK2	CeRNA	Reversing	133
AC073284.4	Paclitaxel	miR‐18b‐5p/DOCK4	CeRNA	Reversing	134

## Abbreviations

ABC: ATP-binding cassette
AGO2: argonaute 2
AICD: activation-induced cell death
ASOs: antisense oligonucleotides
ATM: ataxia-telangiectasia mutated
BC: breast cancer
BCSCs: breast cancer stem cells
cAMP: cyclic AMP
CBP: CREB-binding protein
Cdc37: cell division cycle 37
CDKs: cyclin-dependent kinases
ceRNAs: competing endogenous RNAs
CTLs: cytotoxic T lymphocytes
DBT: digital breast tomosynthesis
DOCK4: dedicator of cytokinesis protein 4
EMT: epithelial-mesenchymal transition
ER: estrogen receptor
exRNAs: extracellular RNAs
FTH1P3: ferritin heavy chain 1 pseudogene 3
GPCR: G-protein-coupled receptor
HBO1: human acetylase binding to ORC1
HER2: human epidermal receptor 2
Hh: hedgehog signaling
HR: homologous recombination
HSP90: heat-shock protein 90
iPSCs: induced pluripotent stem cells
LAR: luminal androgen receptor
LINK-A: long intergenic non-coding RNA for kinase activation
LNAs: locked nucleic acids
lncRNAs: long noncoding RNAs
MANCR: mitotically-associated long noncoding RNA
MDR: multidrug resistance
MECP2: methyl CpG-binding protein 2
miRNAs: microRNAs
MRI: magnetic resonance imaging
MSCs: mesenchymal stem cells
NEAT1: nuclear paraspeckle assembly transcript 1
NHEJ: nonhomologous end joining
OS: overall survival
PAR: polymeric adenosine diphosphate ribose
PARP: poly (ADP-ribose) polymerase
PCA3: Prostate Cancer gene 3
PD-1: programmed cell death protein-1
PFS: progression-free survival
PKA: protein kinase A
PLC: peptide-loading complex
PR: progesterone receptor
RHA/DHX9: RNA helicase A
RISC: RNA-induced silencing complex
SAM: synergistic activation mediator
SERDs: selective estrogen receptor degraders
SERMs: selective estrogen receptor modulators
SILVER-seq: Small Input Liquid Volume Extracellular RNA Sequencing
siRNAs: small interfering RNAs
TCL6: T-cell leukemia/lymphoma 6
T-DM1: trastuzumab emtansine
TMPO-AS1: thymopoietin antisense transcript
TNBC: triple-negative breast cancer
TRF2: telomeric repeat-binding factor 2
AICD: activation- induced cell death
CBP: CREB-binding protein
CDKs: cyclin-dependent kinases
ceRNAs: competing endogenous RNAs
CTLs: cytotoxic T lymphocytes
DOCK4: dedicator of cytokinesis protein 4
ER: estrogen receptor
FTH1P3: ferritin heavy chain 1 pseudogene 3
GPCR: G-protein-coupled receptor
HER2: human epidermal receptor 2
LINK-A: long intergenic non-coding RNA for kinase activation
lncRNAs: long noncoding RNAs
MECP2: methyl CpG-binding protein 2
NEAT1: nuclear paraspeckle assembly transcript 1
PARP: poly (ADP-ribose) polymerase
PLC: peptide-loading complex
TMPO-AS1: thymopoietin antisense transcript
TRF2: telomeric repeat-binding factor 2
UCA1: urothelial carcinoma associated 1
